# Antimicrobial activities of the methanol extract, fractions and compounds from *Ficus polita *Vahl. (Moraceae)

**DOI:** 10.1186/1472-6882-11-6

**Published:** 2011-01-26

**Authors:** Victor Kuete, Justin Kamga, Louis P Sandjo, Bathelemy Ngameni, Herve MP Poumale, Pantaleon Ambassa, Bonaventure T Ngadjui

**Affiliations:** 1 Department of Biochemistry, Faculty of Science, University of Dschang, Cameroon; 2 Department of Organic Chemistry, Faculty of Science, University of Yaoundé I, Cameroon; 3 Department of Pharmacy and Traditional Pharmacopoeia, Faculty of Medicine and Biomedical Science, University of Yaoundé I, Cameroon

## Abstract

**Background:**

Many plants of the family Moraceae are used in the treatment of infectious diseases. *Ficus polita *Vahl., an edible plant belonging to this family is used traditionally in case of dyspepsia, infectious diseases, abdominal pains and diarrhea. The present work was designed to assess the antimicrobial activity of the methanol extract from the roots of *F. polita *(FPR), as well as that of its fractions (FPR1-5) and two of the eight isolated compounds, namely euphol-3-*O*-cinnamate (**1**) and *(E)-*3,5,4'-trihydroxy-stilbene-3,5-*O-β-D*-diglucopyranoside (**8**).

**Methods:**

The liquid microdilution assay was used in the determination of the minimal inhibitory concentration (MIC) and the minimal microbicidal concentration (MMC), against seven bacterial and one fungal species.

**Results:**

The results of the MIC determination showed that the crude extract, fractions FPR1, FPR2 and compound **8 **were able to prevent the growth of the eight tested microorganisms. Other samples showed selective activity. The lowest MIC value of 64 μg/ml for the crude extract was recorded on 50% of the studied microbial species. The corresponding value for fractions of 32 μg/ml was obtained on *Salmonella typhi*, *Escherichia coli *and *Candida albicans ATCC strains. *The MIC values recorded with compound **8 **on the resistant *Pseudomonas aeruginosa *PA01 strain was equal to that of chloramphenicol used as reference antibiotic.

**Conclusion:**

The obtained results highlighted the interesting antimicrobial potency of *F. polita *as well as that of compound **8**, and provided scientific basis for the traditional use of this taxon in the treatment of microbial infections.

## Background

According to the World Health Organization (WHO), infectious diseases is the first cause of death worldwide with more that 50% of the death appearing in tropical countries. In the developing countries, treatment of such diseases is complicated not only because of the occurrence of resistant microorganisms to the commonly used antibiotics, but also because of the low income of the population, which drastically reduce their accessibilities to appropriate drugs. It is reported that about 80% of the world population is dependent (wholly or partially) on plant-based drugs [1]. Scientific experiments on the antimicrobial properties of plant components were first documented in the late 19^th ^century [2]. Naturally occurring antimicrobials can be derived from plants, animal tissues, or microorganisms [3]. The shortcomings of the drugs available today propel the discovery of new pharmacotherapeutic agents in herbal medicine [4]. Amongst the medicinal plants investigated in our research team, the family Moraceae is largely represented. Some of the plants of the genus *Ficus, Morus, Treculia, Dorstenia *, also belonging to this family, were previously reported for their antimicrobial activities [5-11]. In our continuous search on medicinal plants of the family Moraceae, we focused herein on *Ficus polita *Vahl. *F. polita *is an edible plant growing in lowland rainforest and gallery forest (West and Central Africa), coastal and dry forest (East and Southern Africa), up to an altitude of 1200 m. The edible fruits are chewed for dyspepsia, while leaves (also edible) or bark and roots infusions are used in the treatment of infectious diseases, abdominal pains and diarrhea [12,13]. The phytochemical investigation of this taxon [14] revealed the presence of a cerebroside named politamide, sitosterol 3-*O*-β-D-glucopyranoside, betulinic acid, stigmasterol and lupeol. The methanol and dichloromethane extract from the stem exhibited anti-inflammatory activities [15]. Water extract from this plant also showed anti-HIV activity through the inhibition of HIV-1 reverse transcriptase activity [16]. Extracts from the leaves of *F. polita *also exhibited antimalarial action against *Plasmodium falciparum *cultured *in vitro *[17]. The present work was therefore undertook to evaluate the antimicrobial activities of the extract, fractions and compounds from the roots of this plants, and to identify some of it's active components.

## Methods

### Plant material

The roots of *Ficus polita *Vahl., were collected in Yaounde, Center region of Cameroon in May 2007. The plant was identified at the National Herbarium (Yaounde, Cameroon) where a voucher specimen was deposited under the reference number 39955/HNC.

### Extraction and purification

**Figure 1:**
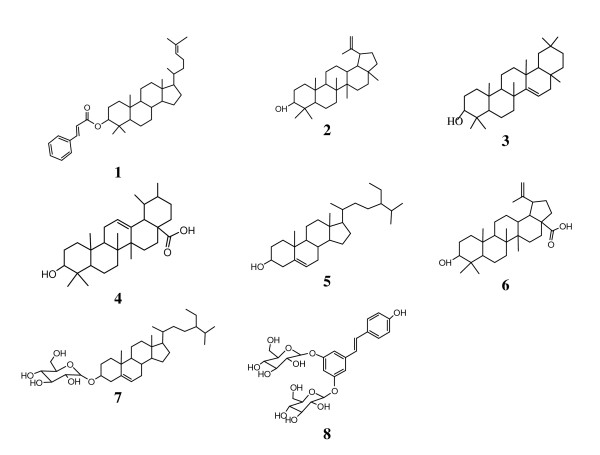
**Chemical structures of compounds isolated from the roots of *Ficus polita***. **1**: euphol-3-*O*-cinnamate; **2**: lupeol; **3**: taraxar-14-ene; **4**: ursolic acid; **5**: *ß*-sitosterol, **6**: betulinic acid; **7**: sitosterol-3-O-*ß-D*-glucopyranoside; **8**: *(E)-*3,5,4'-trihydroxy-stilbene-3,5-*O-β-D*-diglucopyranoside.

The air-dried and powdered root (3.1 kg) was soaked in 10 L of methanol for 48 h, at room temperature. The methanol extract was concentrated under reduced pressure to give 216 g of a brown residue that constituted the crude extract (FPR). Part of FPR (200 g) was submitted to silica gel 60 (0.04-0.063 mm, 200 g) vacuum flash chromatography using as eluent, hexane (Hex), hexane-ethyl acetate (Hex-EtOAc) mixture of increasing polarity, and methanol (MeOH). This was conducted in accordance to the previously reported procedure [14]. Five fractions were obtained, FPR1 (from Hex, 23 g), FPR2 (Hex-EtOAc 75%; 18 g), FPR3 (Hex-EtOAc 50%, 14 g), FPR4 (EtOAc, 12 g) and FPR5 (MeOH, 19 g). A part from FPR3, other fractions, upon antimicrobial assay were subjected to further purification. FPR1 (20 g) was column chromatographed using silica gel 60 (65 g) and Hex-EtOAc gradient as eluent. One hundred and forty seven (147) fractions of 50 ml each were collected. Sub-fractions 2-7 obtained with Hex-EtOAc 2.5% and combined on the basis of TLC analysis afforded compound **1 **[18]. Sub-fraction 34-40 (Hex-EtOAc 10%) yielded compound **2 **[19]. Sub-fractions 51-57 (Hex-EtOAc 15%) yielded compound **3 **[20], while sub-fractions 76-81 obtained in Hex-EtOAc 22.5% gave compound **4 **[21]. FPR2 was subjected to column chromatography (CC) similarly to FPR1 and 76 fractions of 50 ml each were collected. Sub-fractions 26-33 eluted with Hex-EtEtOAc 15% yielded compound **5 **[22] while sub-fractions 38-41 eluted with Hex-EtEtOAc 20% afforded compound **6 **[23]. FPR4 was subjected to CC similarly to FPR1 and 2 using CH_2_Cl_2_-MeOH gradient as eluent and 107 sub-fractions were collected. Sub-fractions obtained in CH_2_Cl_2_-MeOH 5% afforded compound **7 **[22]. FPR5 was also subjected to CC similarly to FPR4 and 93 sub-fractions were collected. Sub-fractions obtained in CH_2_Cl_2_-MeOH 10% afforded compound **8 **[22]. The chemical structures of the isolated compounds are illustrated in Figure 1.

### General procedure for the identification and characterization of isolated compounds

Aluminum sheet pre-coated with silica gel 60 *F*254 nm (Merck) was used for thin layer chromatography; The spots were visualized using both ultraviolet light (254 and 366 nm) and 50% H_2_SO_4 _spray reagent. NMR spectra were recorded on a Bruker Avance 300 at 300 MHz (1H) and 75 MHz and Bruker Avance 600 at 600 MHz (^1^H) and 150 MHz (^13^C), with the residual solvent peaks as internal references. The melting point (m.p.) were determined using a Kofler microhot stage apparatus. Mass spectra were recorded with API QSTAR pulsar mass spectrometer. The structures of the compounds were confirmed by comparing with reference data from available literature.

### Antimicrobial assays

#### Microbial strains and culture media

The studied microorganisms included reference strains of *Providencia smartii *(ATCC29916)*, Pseudomonas aeruginosa *(PA01)*, Klebsiella pneumoniae *(ATCC11296)*, Staphylococcus aureus *(ATCC25922)*, Salmonella typhi *(ATCC6539)*, Escherichia coli *(ATCC8739 and AG100)*, Candida albicans *(ATCC 9002) obtained from the American Type Culture Collection. They were maintained on agar slant at 4˚C and sub-cultured on a fresh appropriate agar plates 24 h prior to any antimicrobial test. Nutrient Agar and Sabouraud Glucose Agar were used for the activation of bacteria and fungi respectively. The Mueller Hinton Broth (MHB) was used for the MIC and MMC determinations. The Mueller Hinton Agar (MHA) was also used for the determination of the MMC on these species.

#### Chemicals for antimicrobial assay

Chloramphenicol (Sigma-Aldrich, St. Quentin Fallavier, France) and Nystatin (Sigma-Aldrich) were used as reference antibiotics (RA) respectively against bacteria and *Candida albicans*. *p*-Iodonitrotetrazolium chloride (INT, Sigma-Aldrich) was used as microbial growth indicator.

#### MIC and MMC determinations

The MIC determinations on bacteria and *C. albicans *were conducted using rapid XTT colorimetric assay according to described methods [24,25] with some modifications. Briefly, the test sample was first of all dissolved in 10% (v/v) DMSO/MHB to give a final concentration of 512 μg/ml and serially diluted twofold to obtain the concentration ranges (4-512 μg/ml for the crude extract, fractions and isolated compounds, and 1-128 μg/ml for chloramphenicol and nystatin used as RA). 100 μl of each concentration was added in a well (96-well microplate) containing 95 μl of MHB and 5 μl of inoculum (standardized at 1.5×10^6 ^CFU/ml by adjusting the optical density to 0.1 at 600 nm SHIMADZU UV-120-01 spectrophotometer) [26]. The final concentration of DMSO in the well was less than 3% (preliminary analyses with 3% (v/v) DMSO do not alter the growth of the test organisms). The negative control well consisted of 195 μl of MHB and 5 μl of the standard inoculum [27]. The plates were covered with a sterile plate sealer, then agitated to mix the contents of the wells using a plate shaker and incubated at 37°C for 24 h. The assay was repeated three times. The MIC of samples was detected following addition (40 μl) of 0.2 mg/ml *p*-iodonitrotetrazolium chloride and incubation at 37°C for 30 min [24,25]. Viable microorganisms reduced the yellow dye to a pink colour. MIC was defined as the lowest sample concentration that prevented this change and exhibited complete inhibition of bacterial growth. For the determination of MMC, a portion of liquid (5 μl) from each well that showed no change in colour was plated on MHA and incubated at 37°C for 24 h. The lowest concentration that yielded no growth after this sub-culturing was taken as the MMC [8].

## Results and discussion

The chemical structures of the isolated compounds were established using spectroscopic analysis, especially, NMR spectra in conjunction with 2D experiments, COSY, HMQC, HMBC, and direct comparison with published information and with authentic specimens obtained by our research group for some cases. The compounds isolated from the roots of *F. polita *(Figure 1) were identified as euphol-3-*O*-cinnamate C_39_H_56_O_2 _(**1**; 14 mg; Mw : 556; m.p. 110-111°C) [18], lupeol C_30_H_50_O (**2**; 44 mg; Mw : 426; m.p. 215-216°C) [19], taraxar-14-ene C_30_H_50_O_1 _(**3**; 13 mg; Mw: 426; m.p. 211-213°C) [20], ursolic acid C_30_H_50_O_3 _(**4**; 18 mg; Mw : 456; m.p. 284-285°C) [21], *ß*-sitosterol C_29_H_50_O (**5**; 220 mg; Mw : 414; m.p. 277-278°C) [22], betulinic acid C_30_H_48_O_3_(**6**; 16 mg; Mw : 456; m.p. 295-297°C) [23], sitosterol 3-O-*ß-D*-glucopyranoside C_35_H_60_O_6 _(**7**; 25 mg; Mw: 576; amorphous powder) [22] and *(E)-*3,5,4'-trihydroxy-stilbene-3,5-*O-β-D*-diglucopyranoside C_26_H_32_O_13 _(**8**; 30 mg; Mw : 576; amorphous powder) [22]. However, the isolation of compounds **2**, **4, 6 **and **7 **from this plant was reported [14]. Using previously described protocol [14] for the purification of *F. polita*, four additional known compounds (**1**, **3**, **5 **and **8**) were isolated from the roots extract. The isolated compounds included non polar terpenoids as well as polar compounds such *(E)-*3,5,4'-trihydroxy-stilbene-3,5-*O-β-D*-diglucopyranoside, justifying the use of methanol as a good extraction solvent of bioactive metabolites from medicinal plants. This extract, fractions as well as compounds **1 **and **8 **were tested for their antibacterial activities and against *C. albicans. *The results are reported in Tables 1 and 2.

**Table 1:** MIC (μg/ml) of the crude extract, fractions and compounds isolated from the roots of *F. polita *and reference antibiotics on the studied microbial species.

**Tested samples**^**a**^	**Microorganisms, strains and MIC (μg/ml)**^**b**^							
	
	*P. smartii*	*P. aeruginosa*	*K. pneumoniae*	*S. aureus*	*S. typhi*	*E. coli*		*C. albicans*
							
	ATCC29916	PA01	ATCC11296	ATCC25922	ATCC6539	ATCC8739	AG100	ATCC 9002
FPR	128	128	128	64	64	64	256	64
FPR1	64	64	128	64	32	32	128	32
FPR2	256	128	256	256	256	128	256	64
FPR3	-	-	-	-	-	512	-	-
FPR4	512	-	512	-	-	512	-	-
FPR5	256	256	512	128	256	256	-	128
**1**	-	512	-	512	512	256	-	512
**8**	256	64	128	64	64	64	256	128
**RA**	32	64	4	4	4	4	4	16

^a^The tested samples were compounds **1**: euphol 3-*O*-cinnamate; **8**: *(E)-*4'-hydroxystilbene-3,5-*O-β-*diglucopyranoside and chloramphenicol (for bacteria) and nystatin (for *C. albicans*) used as the reference antibiotics (RA); the crude extract (FPR) and fractions (FPR1 to 5) from *F. polita *roots;

^b^The tested microorganisms were *Providencia smartii (P. smartii); Pseudomonas aeruginosa (P. aeruginosa); Klebsiella neumonia (K. pneumonia); Staphylococcus aureus (S. aureus); Salmonella typhi (S. typhi); Escherichia coli (E. coli); Candida albicans (C. albicans). *(-): MIC > 512 μg/ml

**Table 2:** MMC (μg/ml) of the crude extract, fractions and compounds isolated from the roots of *F. polita *and reference antibiotics on the studied microbial species.

**Tested samples**^**a**^	**Microorganisms, strains and MIC (μg/ml)**^**b**^							
	
	*P. smartii*	*P. aeruginosa*	*K. pneumoniae*	*S. aureus*	*S. typhi*	*E. coli*		*C. albicans*
							
	ATCC29916	PA01	ATCC11296	ATCC25922	ATCC6539	ATCC8739	AG100	ATCC 9002
FPR	256	256	256	128	256	128	512	128
FPR1	128	128	256	128	64	64	128	64
FPR2	512	512	512	512	512	256	512	256
FPR3	nd	-	-	-	-	>512	nd	nd
FPR4	>512	-	>512	-	-	>512	nd	nd
FPR5	512	512	>512	128	512	512	nd	128
**1**	nd	>512	nd	>512	>512	>512	nd	>512
**8**	512	256	256	128	128	128	512	256
**RA**	64	128	8	8	8	8	8	32

^a^The tested samples were compounds **1**: euphol 3-*O*-cinnamate; **8**: *(E)-*4'-hydroxystilbene-3,5-*O-β-*diglucopyranoside and chloramphenicol (for bacteria) and nystatin (for *C. albicans*) used as the reference antibiotics (RA); the crude extract (FPR) and fractions (FPR1 to 5) from *F. polita *roots;

^b^The tested microorganisms were *Providencia smartii (P. smartii); Pseudomonas aeruginosa (P. aeruginosa); Klebsiella pneumoniae (K. pneumoniae); Staphylococcus aureus (S. aureus); Salmonella typhi (S. typhi); Escherichia coli (E. coli); Candida albicans (C. albicans). *(-): not determined because MIC value was not detected up to 512 μg/ml; (nd): not determined; >512: value above 512 μg/ml.

The MIC results summarized in Table 1, showed that the crude extract, fractions FPR1, FPR2 and compound **8 **were able to prevent the growth of all the eight tested microorganisms. Other samples showed selective activity. The lowest MIC value of 64 μg/ml for the crude extract was recorded on four of eight (50%) studied microbial species. The corresponding value for fractions (32 μg/ml) was obtained on *Salmonella typhi*, *Escherichia coli and Candida albicans ATCC strains. *Such activity can be considered as important, when considering the threshold MIC value (100 μg/ml) for plant extracts with significant activity [28]. Nonetheless, the corresponding cutoff point for compounds (10 μg/ml), is not acheived with the tested compounds. However compound **8 **exhibited moderate activities [28], the MICs below 100 μg/ml being recorded on three of the eight microorganisms tested. Taking in account the fact that *F. polita *is and edible plant, its seems reasonable to consider a more flexible stringent criteria indicating that extracts with MIC values below 8 mg/ml [29] are considered to possess some antimicrobial activity and natural products with MIC values below 1 mg/ml are considered noteworthy [30,31]. Therefore, the activity recorded therefore with the extract, some of the fractions and compounds **8 **could be considered as important, highlighting the antimicrobial potency of *F. polita. *The MIC values recorded with compound **8 **on the resistant *P. aeruginosa *PA01 was equal to that of chloramphenicol used as reference antibiotic, confirming this hypothesis. The results of Table 2 showed detectable MMC values for some of the studied samples on the tested microbial strains. When analysing carefully the MIC and MMC results for the crude extract, fractions FPR1 and 2, compounds **8**, it can be noted that MMC/MIC ratios lower than 4 were obtained with most of the studied samples, suggesting that killing effects could be expected [32]. To the best of our knowledge, the activity of this plant, compounds **1 **and **8 **on the microorganisms studied in the present work is being reported for the first time. Compounds with enough evidence of their antimicrobial microbial activities such lupeol [33], betulinic acid [34], ursolic acid [35], *β*-sitosterol, sitosterol-3-*O-β-D*-glucopyranoside [36,37], were not tested again in the present work. However, compounds such as lupeol exhibited moderate inhibitory effect against *E. coli *and *Mycobacterium smegmatis *[33]. Nonetheless, these terpenoid-like compounds mostly exhibited poor or moderate activities. Their presence as well as that of Euphol 3-*O*-cinnamate and *(E)-*4'-hydroxystilbene-3,5-*O-β-*diglucopyranoside, and possible synergistic activities, can explain the good antimicrobial activity observed with the crude extract of *F. polita *and fractions. Finally, the overall activities of this plant might be due to the presence of several antimicrobial compounds with moderate activities.

## Conclusion

The results of this study provide informative data for the use of the crude extract from *Ficus polita *against bacterial microbial infections.

## Competing interests

The authors declare that they have no competing interests.

## Authors' contributions

VK and JK carried out the study and wrote the manuscript; LPS, BN and PA participate to structural elucidation of compounds. BTN supervised the work and the manuscript writting. All authors read and approved the final manuscript.

## Pre-publication history

The pre-publication history for this paper can be accessed here:

http://www.biomedcentral.com/1472-6882/11/6/prepub
